# Source Coding Options to Improve HEVC Video Streaming in Vehicular Networks

**DOI:** 10.3390/s18093107

**Published:** 2018-09-14

**Authors:** Pedro Pablo Garrido Abenza, Manuel P. Malumbres, Pablo Piñol, Otoniel López-Granado

**Affiliations:** Department of Physics and Computer Architecture, Miguel Hernández University, 03202 Elche, Spain; mels@umh.es (M.P.M.); pablop@umh.es (P.P.); otoniel@umh.es (O.L.G.)

**Keywords:** vehicular networks, video delivery, QoS, QoE, HEVC, OMNeT++, Veins, SUMO

## Abstract

Video delivery in Vehicular Ad-hoc NETworks has a great number of applications. However, multimedia streaming over this kind of networks is a very challenging issue because (a) it is one of the most resource-demanding applications; (b) it requires high bandwidth communication channels; (c) it shows moderate to high node mobility patterns and (d) it is common to find high communication interference levels that derive in moderate to high loss rates. In this work, we present a simulation framework based on OMNeT++ network simulator, Veins framework, and the SUMO mobility traffic simulator that aims to study, evaluate, and also design new techniques to improve video delivery over Vehicular Ad-hoc NETworks. Using the proposed simulation framework we will study different coding options, available at the HEVC video encoder, that will help to improve the perceived video quality in this kind of networks. The experimental results show that packet losses significantly reduce video quality when low interference levels are found in an urban scenario. By using different INTRA refresh options combined with appropriate tile coding, we will improve the resilience of HEVC video delivery services in VANET urban scenarios.

## 1. Introduction

Among the potential applications that may be supported by Vehicular Ad-hoc NETworks (VANETs), video delivery is one of the most resource-demanding. Several application scenarios may require video delivery services in either on-demand or real-time live video streaming, using unicast, multicast or broadcast communications. We may find scenarios where video delivery is required, like the ones related to accidents/rescue assistance, V2X real-time video, context-aware video broadcasts, security surveillance services, driving assistance, etc. However, multimedia streaming over VANETs is a very challenging issue mainly due to the high mobility of vehicles, bandwidth limitations, and the high loss rates typically found in wireless communications. In addition, video transmission requires a high bandwidth with a bounded packet delay, especially when real-time restrictions are mandatory. So, when video suffers from packet losses and/or highly variable packet delays, the user perceived video quality is seriously reduced.

In this work, we analyze the performance of video delivery in a typical VANET urban scenario by means of a simulation framework named Video Delivery Simulation Framework over Vehicular Networks (VDSF-VN) [1], which allows us to model in detail the different actors involved in a video streaming session. VDSF-VN works with the OMNeT++ simulator [2], together with the Veins (VEhicles In Network Simulation) framework [3] to conduct the network simulations, and with SUMO (Simulation of Urban MObility) [4], as the vehicular traffic simulator.

Inside VDSF-VN, the selected source video sequence is encoded with the High Efficiency Video Coding (HEVC) standard [5]. In particular, we have developed a tuned version of the HEVC reference software HM (HEVC Test Model) [6] that it is able to seamlessly work with OMNeT++/Veins/SUMO simulation tools. Also, it has been necessary to develop several software modules, such as a packetizer/depacketizer tool, an RTP packet trace module, etc. All of these software modules are governed from a graphical application, named GatcomVideo, that will significantly improve the usability and automation of the proposed VDSF-VN framework.

Although the simulation framework provides a lot of useful performance metrics (end-to-end delay, goodput, packet delivery ratio, etc.), our main performance metric is the video quality delivered to the user, since it will be used to determine the minimum quality levels that a video delivery application should provide. After analyzing several simulation runs we notice that the received video quality becomes unacceptable when the first interferences appear in the network. So, we proceed to study some coding options implemented in the HEVC video encoder, in order to improve the final video quality delivered to the user.

These options are based on INTRA refresh and tile-based video coding techniques. On the one hand, INTRA refresh technique will help to stop the error propagation produced by a single packet loss in the prediction process of future (next) video frames. So, we have analyzed the performance of several INTRA refresh approaches in order to determine the ones which deliver a higher video quality to the user with an acceptable bit rate overhead. On the other hand, TILE-based coding allows partitioning each video frame in blocks (Tiles), which are completely independent regarding coding/decoding. This may be useful to mitigate the impact of packet losses when compared with no-tiling approaches. When no-tiling approaches (the default coding option) are used, an encoded frame is typically fragmented into several network packets. To decode the frame, all those packets need to be received. If just one packet is lost, the frame is undecodable although the rest of packets had been correctly received. If tiling is used, a frame can be partially decoded (not completely lost) improving the final video quality at the same packet error rate.

A thorough experimental process was developed in order to identify the above mentioned HEVC coding options which offer the best video quality to the user. The simulation results obtained from the experimental tests will show the behavior of the HEVC coding options under different network impairment degrees. This knowledge will provide us with the ability to decide which level of INTRA refreshing is required and how many tiles per frame should be used to guarantee the minimum video quality required by the video delivery application.

The rest of the paper is structured as follows. Section 2 shows some related works, specifically some techniques and simulation tools used in the literature for improving the video quality perceived by the final user in wireless networks. In Section 3, we briefly describe the video delivery simulation framework we have used in this work. Section 4 describes the simulation methodology, the VANET scenario and the video sequence used. The simulation results of the experimental tests are presented and discussed in Section 5. Finally, conclusions are drawn in Section 6, along with references to future work.

## 2. Related Works

Many different techniques have been proposed in the literature to improve video streaming over not reliable medium like the wireless channel, in general, and VANETs, in particular [7].

Error Concealment (EC) methods try to minimize the impact of packets loss in the perceived quality of the received video sequence [8,9,10]. If any part of an encoded frame is missing, then the entire frame cannot be decoded. These EC methods try to predict the missing parts and correct them by exploiting spatial similarities of the nearby areas, or temporal redundancies in the video sequence [11]. Spatial prediction can be only used for small areas [12], whereas the temporal prediction can replace a whole missing frame, replacing it with a previously received frame. This is known as ’Frame Copy Concealment’ [13]. To make an estimation of the contents of a missing area, different techniques based on Motion Vectors (MV) information can also be used.

Error Resilience (ER) is a group of techniques which aim to provide robustness to video sequences, which are applied at the sender side. One of these is the Feedback Channel (FC), in which the receiver can request the retransmission of some missing part [14]. Other methods make use of substreams, such as Multiple Description Coding (MDC) [15] or Layered Coding (LC).There has been a lot of research done to protect video sequences with Forward Error Correction (FEC), also known as Channel Coding [16]. This technique inserts redundant data, called Error Correction Codes (ECCs), into the encoded video. These ECCs can be used for detecting missing data on the receiver side, and even, to recover them without retransmission, which is not feasible in some scenarios like real-time applications (e.g., live video streaming) [17]. RaptorQ codes is an example of this kind of mechanism [18]. FEC can be used in all kind of networks, including VANETs [19].

Another group of ER techniques are the so-called ’intra refresh’ methods. They encode the bit stream in such a way that it prevents fast quality degradation in the presence of data losses. An inter-coded frame, a frame which it is predicted using other frames as references, although correctly received, can be affected by a reference frame with errors, and it can propagate the error to other inter-frames which use it as reference. A proper insertion of intra-coded frames together with an adequate selection of reference frames may increase the error resilience of an encoded bit stream. So, defining appropriate INTRA refresh encoding modes will make bit streams more robust. There are several works in the literature that exploit these techniques to improve video robustness like the ones found in [20,21,22].

To evaluate the performance of source coding techniques we need a simulation framework to analyze their effects in the final video quality experienced by a VANET user. To measure video quality we will use the Peak Signal-to-Noise Ratio (PSNR) metric. So, the error protection of our encoder will be based on those encoding options which provide a better received video quality, taking into account the bit rate overhead which they typically provide.

There are several simulation frameworks proposed in the literature that follow our requirements, like EvalVid [23], which allows the video quality evaluation of MPEG4 video transmitted over a real or simulated communication network. Besides measuring several metrics of the underlying network, like loss rate, delay, and jitter, they also support a video quality evaluation of the received video based on the frame-by-frame PSNR calculation. Although no specific network simulator is proposed, authors argue that theoretically any simulator could be used. In this sense, several works have extended EvalVid to include a network simulator. For example, in [24] EvalVid is integrated with ns-2. In [25] authors developed a tool named QoE Monitor, which is based on the EvalVid architecture and consists of a new module for the ns-3 simulator. Beside this, this tool can be extended with current video coding standards. In [26] a simulation framework named Mobile Multi-Media Wireless Sensor Networks (M3WSN) is presented, which is based on EvalVid too. This framework uses the OMNeT++ simulator and Castalia framework [27].

Another example is HEVStream [28], which is a realistic testbed environment that aims to evaluate HEVC video streaming under different conditions. However, it is hardware-based and we need to use the simulation technique because our target is to evaluate video streaming in vehicular networks.

Despite of the different existing video frameworks (most of them based on the initial EvalVid approach), none of them allows the transmission and quality evaluation of video sequences with the combination of the OMNeT++ simulator, the Veins framework and the SUMO traffic mobility model for vehicular networks. Some analyzed frameworks lack of an updated video codec module, being not trivial to change it with another one, because the packetizer needs to be properly adapted to the intrinsic features of the target bit stream syntax. Also, node mobility models are too simple in most approaches to fit with the particularities of vehicular networks (roads, streets, lanes, stops, traffic lights, etc.). In addition, finally, the different modules of the framework need to be completely integrated in order to launch global simulations specifying the detailed configuration of every module, and performing, on the fly, all the processes, from the video encoding at source node to the decoding process at the receiver end, passing through the network simulation of the video delivery in a realistic vehicular network scenario. These aspects have motivated us to develop the VDSF-VN [1] simulation framework which it is especially suited for vehicular networks. VDSF-VN is the simulation framework that we use in this work, so we provide a brief introduction of it in the following section.

## 3. Simulation Framework: VDSF-VN

**Figure 1:**
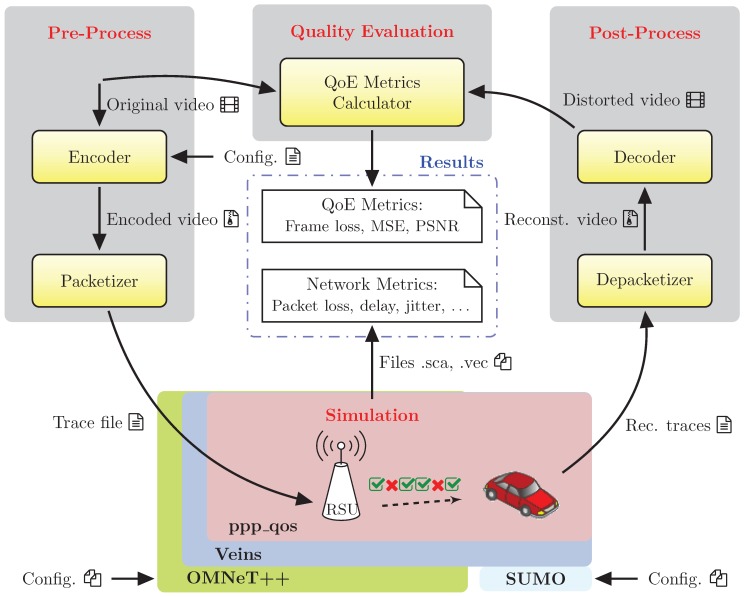
Workflow of VDSF-VN.

In Figure 1, we show the different elements that form the VDSF-VN simulation framework architecture. Some of those elements have to do with the video coding and decoding processes (encoder, packetizer, etc.), whereas some others are related to the simulation process (OMNeT++, Veins and SUMO). Apart from these, a graphical application named “GatcomVideo” was developed which acts as a front-end that manages the whole process. Finally, in order to build the simulation network and configuration files, the “GatcomSUMO” [29] application was also developed to ease all the configuration duties by means of a Graphical User Interface (GUI).

The VDSF-VN framework coordinates and manages all the necessary tasks involved with this kind of experiments, such as, source video encoding, trace files generation, video decoding tasks, running the simulation sequences and obtaining the desired plots from the simulation statistic results. VDSF-VN was also designed to be multithread-aware, so several tasks or simulation runs may be launched in parallel depending on the available hardware resources.

As depicted in Figure 1, all of these tasks can be structured in three main steps: (1) pre-process, (2) simulation, and (3) post-process.

1. Pre-process: This step is in charge of encoding the desired video sequences and then perform the Real-time Transmission Protocol (RTP) packetization of the encoded bit stream and generate the corresponding video trace file. This step is a time-consuming task, because the video encoding requires a great amount of computation and the video sequence may be encoded with different configuration parameter sets.
2. Simulation: This step includes the actions to prepare the network scenario and run the simulations with OMNeT++, Veins and SUMO. The trace file from the pre-process step is loaded by the video servers to simulate the video broadcasting. During the simulation, the client nodes write the correctly received video packets into an output trace file, which will be used in the post-process step. This is done in an OMNeT++ project named “ppp_qos”, which references the original Veins project, but it is implemented in a separate project for convenience. This project includes the “TraCIDemo11p” application module provided in Veins, which has been extended with: (a) the support of HEVC video trace files, (b) statistics at application level (load, goodput, end-to-end delay), and (c) statistics related to the mobility of the vehicles (the distance between selected pairs of nodes, the number of neighbors during the simulation, etc.). The “ppp_qos” project also extends the MAC layer with many statistics like the Channel Busy Ratio (CBR), that may be local statistics (i.e., per node), global network statistics or statistics grouped by Access Category (AC).
3. Post-process: This step is taken to get all the statistics defined at different network levels: Physical, MAC, and Application, with the corresponding plots. To compute the application performance metrics like Frame Loss Rate (FLR) and PSNR, the bit stream needs to be conformed with the received packets and then decoded to obtain the reconstructed video which will be rendered to the user. Many works show that PSNR, under some circumstances, does not properly assess the quality perceived by users. In the literature, we can find quality assessment metrics that better correlates with human perceived quality like MS-SSIM [30]. These video quality metrics will be incorporated in future versions of VDSF-VN as an optional feature. However, we will use PSNR metric in this work, as most simulation frameworks also use it.

## 4. Performance Evaluation

This section describes the methodology used in the simulation and the experiments conducted with the proposed VDSF-VN framework for evaluating the video streaming in an urban VANET scenario. Now, we will describe the simulation setup which includes the network scenario, the network simulation parameters, the video source, and the video encoding options.

### 4.1. Scenario Description

**Figure 2:**
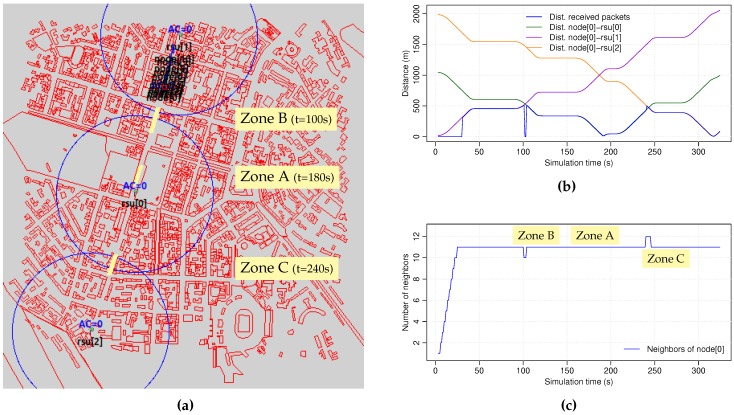
(**a**) Scenario. (**b**) Distance from client to RSUs. (**c**) Number of client neighbors.

The VDSF-VN framework includes a tool, GatcomSUMO [29], that allows defining and configuring the network scenario that will be used in the simulation. Different vehicular network scenarios may be designed: regular (mesh, spider, etc.) or irregular (random). It is also possible to import real urban vehicular scenarios from OpenStreetMap [31], so the desired area of a city may be used as our simulation scenario. In this work, we have used a portion of the city of Kiev (Ukraine), which consists of a square area sized 2000×2000 m^2^ (Figure 2a), downloaded from OpenStreetMap and imported into SUMO. The main simulation parameters are summarized in Table 1.

Three fixed Road Side Units (RSUs) are placed along a central avenue (rsu[0..2]), which act as video servers delivering the same video sequence in a synchronized and cyclic way. The parameters of the network cards are set with the values shown in Table 2. The radio transmission range, which is depicted with a blue circle in Figure 2a, is the default value used in Veins. To get more accurate results, the simulation takes into account the obstacles such as buildings. Specifically, the radio propagation model used is SimpleObstacleShadowing instead of the SimplePathLoss model.

On the other hand, several mobile vehicles are defined, which travel together along the simulation time. The first one is the video client (node[0]), which receives the video sequence sent by the video servers. Then, ten vehicles acting as background traffic nodes follow the first one (node[1..10]), and send packets at different bit rates as background traffic load. To represent different levels of network loads, each background traffic node injects packets with a size of 512 bytes in a sequence of the following rates: {0,12,25,50,75} packets per second (pps). Therefore, on average, the total aggregate background traffic is {0,120,250,500,750} pps, respectively.

All the vehicles follow the same fixed route along the avenue, which is defined as a list of edges. This can be done manually, with the help of some utility provided by SUMO like duarouter, or in a graphical way with the GatcomSUMO application. The maximum speed of the vehicles was fixed to 14 m/s (50 km/h), although their velocity is not constant, as the traffic simulator takes into account intersections, traffic-lights, the presence of other vehicles, etc. SUMO is a microscopic vehicular simulator that slows down or accelerates each vehicle according to the traffic conditions. The mobility model used is the default one in SUMO, that is, a modified version of the Krauss model [32], which is a car following model. Apart from the mobility model (carFollowModel) and the maximum speed (maxSpeed), additional parameters like acceleration (accel), deceleration (deccel), or minimum distance to the vehicle ahead (minGap) are also used.

The simulation time is 340 s, which is time enough for all the vehicles to travel from the beginning to the end of the avenue, receiving the video sequences sent by the three RSUs. Figure 2b shows the distance between the client and the RSUs. Figure 2c shows the number of neighbors of the client node along the entire simulation, showing that all of the background vehicles are within the communication range of the client car all the time. The plot also shows the instant when the node passes through the three selected zones which represent different network situations: an area where the client node has full coverage of one of the RSUs (Zone A), the shadow area between rsu[1] and rsu[0] (Zone B), as well as the overlapping area between rsu[0] and rsu[2] (Zone C).

### 4.2. Video Sequence

The selected video sequence is ’BasketballDrill’, which belongs to the HEVC Common Test Conditions set [6]. As shown in Table 3, it has a resolution of 832×480 pixels and a duration of 10 s. The original sequence is 500 frames long at a rate of 50 frames per second (fps), but it was sub-sampled at 25 fps with a length of 250 frames in order to reduce the required network bandwidth.

This video sequence was encoded with the HEVC reference software HM (HEVC Test Model) [6]. The resulting bit stream consists of a sequence of frames, which can be of three types: I frames (Intra coded), P frames (Predictive coded) and B frames (Bi-directionally predictive coded). A great number of possible encoded bit streams can be generated from a specific raw video sequence depending on the desired HEVC configuration parameters (encoding mode, quantization level, INTRA refreshing, etc.). For example, we can select the main encoding modes provided by the HEVC reference software, namely, All Intra (AI), Low-delay P (LP), Low-delay B (LB), and Random Access (RA), or we can configure other specific encoding modes like the ones that we are going to analyze in this work, shown at Table 4. AI mode is robust to packet loss, as all the frames of the video sequence are encoded as I frames, that is, without using any other frame as reference. On the contrary, LP mode is less robust than AI because it is sensible to packet losses due to the dependencies between frames. In LP mode, the first frame is encoded as an I frame, and the rest of the frames are encoded as P frames, which use previously encoded frames as reference. So, if losses occurs in previous reference frames we cannot correctly predict the content of the current one since we have no information from the past frames. The error produced in the past affects to the encoding performance of future frames (error propagation). However, LP mode is very efficient regarding compression performance because of the use of motion estimation and compensation. This is the reason we propose a set of encoding modes with different levels of periodic updates (INTRA refresh) which is able to efficiently mitigate the error propagation effects without significant performance degradation.

Another coding parameter to define is the Quantization Parameter (QP), which is used to adjust the compression level. A low QP value implies a soft quantization that will result in larger bit streams with higher video quality. In our experiments, the video quality, measured in terms of PSNR, for all the generated bit streams was fixed to the same value by adjusting the compression level (i.e., the QP value) in each case, targeting to a desired PSNR value of approximately 36 dB.

Apart from the compression mode and QP value, the video encoder accepts many other parameters. For example, HEVC allows splitting a frame into several independent regions (tiles or slices). This will be useful for our purposes since it will provide an extra robustness to video delivery when packet losses start to damage the video packet streaming. However, these partitioning modes lower the encoding performance since spatial correlation is limited to the slice/tile domain, and their use requires extra bit stream signaling information [33].

As a result, many combinations of parameters need to be considered. In this work, the selected encoding modes, QP values and number of tiles per frame are the following:

- Encoding modes (×9): {AI, IPIP, I3P, I7P, I15P, IPx, IPx25pctCTU, LPI4, LP} (see Table 4).
- QP values (×1): the QP value used for each encoding mode was fixed to achieve the desired video quality (PSNR ≈ 36 dB) when using 1 tile per frame (see Table 5).
- Number of tiles per frame (×7): seven values were considered to encode each bit stream: {1, 2, 4, 6, 8, 10, 16}. The size of these partitions can be specified with the number of rows and columns (uniform), or with the number of Coding Tree Units (CTUs) per each row and column (non-uniform). In this work, the following uniform tile patterns were considered: {1×1, 1×2, 2×2, 2×3, 2×4, 2×5, 4×4}, respectively.

So, all of these combinations make a total of 63 different bit streams (9×1×7). As it can be appreciated in Table 5, the PSNR value of the original encoded video is approximately the same for all of them, whereas each case has a different bit rate value. As an example, the largest bit stream is achieved with AI mode (3.417 Mbps), whereas the smallest one corresponds to LP (0.959 Mbps); the other combinations are intermediate cases.

Once video encoding is done, we proceed to build a trace file from the encoded bit stream [34]. A trace file is a text file including an ordered list of packets to be transmitted, and is loaded by the video servers before sending it to the clients in the simulation step. Since an encoded frame may be larger than the network Maximum Transmission Unit (MTU), it is necessary to encapsulate it into several packets (packetization). In the trace file, each packet is defined with the following fields: a correlative packet number, the frame type it belongs to (I, P, or B), the playback time (ms), the packet size (bytes), the frame offset of the packet payload, and the total number of fragments of the current frame.

We have modified the HM software to include RTP bit stream packetization [35]. In addition, it was mandatory to modify the HM decoder in order to get the reconstructed video sequence because the original HM decoder crashes when any piece of information is lost, so we have strengthened the decoder to be robust against packet losses.

At last, once the video sequence is reconstructed we compute, among other application metrics, the PSNR value relating to the original video sequence, which is the most commonly used metric for measuring the video quality.

## 5. Simulations Results

The purpose of this paper is to find out how different encoding modes affect the video delivery in vehicular networks, that is, to discover which of the generated bit streams achieves the best trade-off between reconstructed video quality and resulting bit rate. So, we start with an evaluation of the different INTRA refresh modes without using tile partitioning (i.e., only one tile per frame). Then we analyze the effect of increasing the number of tiles per frame with the All-Intra (AI) coding mode. In addition, finally we will see in action both INTRA refresh modes and the use of tiles to evaluate their overall behavior on error resilience, video quality and coding bit rate.

Although there are much more metrics available, we have selected the following application layer metrics:

- Goodput (GP): the total number of received video packets at the client application layer divided by the application runtime (i.e., from the sending time of the first packet until the time when the last packet is received).
- End-to-End (E2E) Delay: the average time each packet requires in order to be delivered to the destination application (running at a particular node).
- Jitter: the average E2E delay variation between two consecutive packets received at the destination node.
- Packet Delivery Ratio (PDR): the relation between the total number of video packets received by the client with respect to the video packets sent by the server. As all the video servers (RSUs) transmit the same video sequence in a synchronized and cyclic way, in this work, only the number of packets sent by the associated RSUs is considered.
- Packet Loss Ratio (PLR): the percentage of lost packets out of the total number of packets of the video sequence.
- Tile Loss Ratio (TLR): the percentage of tiles that have not been completely received out of the total number of tiles of the delivered video sequence. In case of using a 1 tile per frame layout (i.e., no-tiling), this value corresponds to the Frame Loss Ratio (FLR).
- Peak Signal-to-Noise Ratio (PSNR): the measure of the objective video quality. The PSNR is computed frame-by-frame for the luminance (Y) component as shown in Equation 1, averaged for all the frames. The Mean Squared Error (MSE) is computed for each frame *n* by averaging the squared intensity differences between each pixel (i,j) of the same original ($Y_{S}$) and distorted ($Y_{D}$) frame, as shown in Equation 2, where $i\in 1..N_{col}$ and $j\in 1..N_{row}$, being $N_{col}$ and $N_{row}$ the width and height of the frames (in pixels).
  (1) $$PSNR{\left(n\right)}_{dB}=20\cdot log_{10}\left(\frac{V_{peak}}{\sqrt{MSE(n)}}\right)$$
  (2) $$MSE\left(n\right)=\frac{1}{N_{col}\cdot N_{row}}\sum_{i=1}^{N_{col}}\sum_{j=1}^{N_{row}}{\left[Y_{S}\left(n,i,j\right)-Y_{D}\left(n,i,j\right)\right]}^{2}$$

The metrics are collected in both scalar (minimum, maximum, mean, etc.) and vector format. Scalar metrics allow to study the overall performance of the network, whereas the vector data allows studying the evolution of such metrics over the entire simulation. To drastically reduce the huge amount of data needed to store the vector data, some of these metrics are also collected in vector format but averaged every second only; in this way, it is possible to analyze the behavior of the network in any zone defined by two given simulation times.

In this work we have analyzed the selected simulation metrics in 3 different fragments of the whole simulation of 10 s in length each:

- Zone A (t = 180..190s): an area where the client node has full coverage of one of the RSUs (rsu[0]).
- Zone B (t = 100..110s): a shadow area between two RSUs (rsu[1] and rsu[0]), where none of them are “visible” for the client node.
- Zone C (t = 240..250s): an area where the coverage of two RSUs are overlapped (rsu[0] and rsu[2]).

Zones B ad C are not shown, as the percentage of lost packets is too high. The location of the RSUs should be adjusted in Zone B, and a handover mechanism should be used in case of overlapping of the transmission ranges (Zone C). Therefore, only results for Zone A are shown below.

### 5.1. INTRA Refresh Coding Modes

In this section the effect of different INTRA refresh modes is analyzed. Since we want to determine the potential of INTRA Refresh alone, only one tile per frame is used, that is, the experiments do not use tile partitioning.

**Figure 3:**
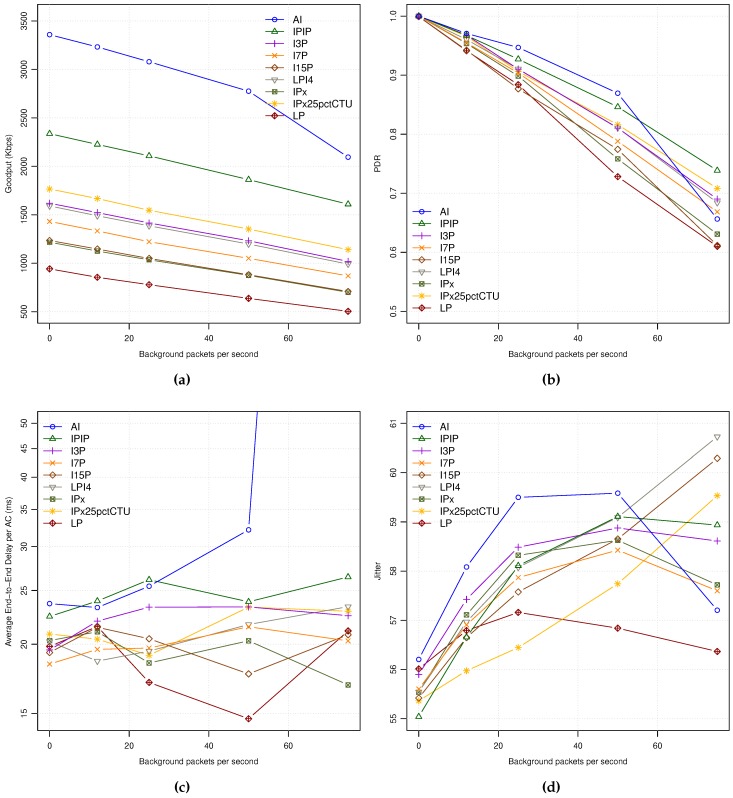
APP statistics for all the encoding modes: (**a**) GP. (**b**) PDR. (**c**) E2E-Delay. (**d**) Jitter.

Figure 3a shows the achieved Goodput for each encoding mode under different background traffic loads. As can be seen, it affects to all the encoding modes in a similar way, proportionally to the different encoding bit rates. Only in the case of the maximum background traffic load (75 pps), the AI mode shows an abrupt descent as the network entered in saturation state, being the rest of coding modes with moderate to high network loads depending on their corresponding bit rates.

As can be seen in Figure 3b, the network Packet Delivery Ratio (PDR) is 1.0 for all the encoding modes when no background traffic is used, meaning that all the packets arrive to their destination. As the background traffic increases, the network PDR decreases for all the encoding modes, being the 50 pps background traffic load a very high network load where more than 15% of packets are lost in all coding modes. Similarly to the previous picture, all the curves are sorted by their consumed bandwidth (see Table 5).

The End-to-End (E2E) Delay shows similar behavior for all the encoding modes while increasing the background traffic (Figure 3c). The AI coding mode suffers from network saturation at the highest background traffic load, achieving average delays of up to 6 s (network saturation). The rest of coding modes show two different behaviors: (a) a soft monotonic delay increase for coding modes with the highest bit rates (i.e., IPIP and I3P), and (b) similar packet delays for the rest of modes that require less bandwidth (bit rate). Among the last coding modes we can observe that the ones with lowest bit rates seem to have less delay as background traffic increases. So, as being the coding modes with lowest bit rates they have a lower probability of sending video packets during the control channel period, reducing the average latency and also the jitter, as explained next.

Regarding Jitter, Figure 3d shows a high average jitter values from 55 milliseconds to more than 60 at high background traffic loads. The main reason for the high jitter observed may be due to the behavior of 802.11p MAC operation, where the time is multiplexed between service and control channels (near the 50% of channel time for each). So, all packets generated during the control channel time period have to wait until the service channel period starts, producing timing distortions in the continuous packet video delivery traffic pattern that seriously affect to Jitter.

**Figure 4:**
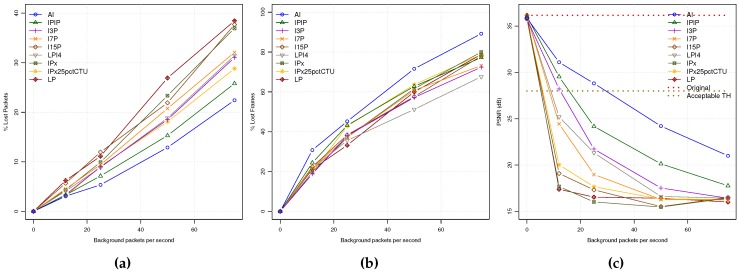
QoE statistics for all the encoding modes: (**a**) PLR. (**b**) FLR. (**c**) PSNR.

In Figure 4a,b, the PLR and FLR application metrics are shown. The PLR follows a behavior coherent with the PDR plot described before: the higher the coding mode bit rate, the lower the PLR value. However, when analyzing the FLR, we can observe that the number of lost frames is inversely proportional to the coding mode bit rates under similar network load conditions. This is due to the bit stream packetization process that produces a higher number of packets per encoded frame for coding modes with higher bit rates. So, the probability of losing one frame is higher, producing more frame losses. In Figure 4c, the PSNR values for all the encoding modes of a video sequence received in Zone A are plotted. As can be seen, the highest video quality is achieved by the AI coding mode followed by IPIP and I3P coding modes. However, as background traffic increases, the PSNR values fall down under unacceptable quality levels (below 28 dBs) for most video contents. On the other hand, LP and IPx have the worst PSNR values; when the first packet losses appear, the video is degraded, being unable to stop error propagation due to their poor INTRA refresh capabilities.

These results demonstrate the benefits of using INTRA refresh encoding modes for improving the reconstructed video quality. However, the price we have to pay for is the increased bit rates, which can cause a greater loss of packets when the network gets saturated.

### 5.2. Tile Partitioning for the All-Intra Coding Mode

After analyzing the INTRA refresh coding modes with their pros and cons, we proceed to study another error resilience technique that may efficiently work together with INTRA refresh. We propose the use of frame partitioning that allow fragmenting each frame into independently encoded/decoded tiles. The idea is to significantly reduce the number of frame losses. In this section we will study the effect of using 1, 2, 4, 6, 8, 10 and 16 tiles per frame with the AI coding mode.

As it was explained in the introduction section, using tiles introduces an overhead in the bit stream as required signaling in form of headers, so higher bit streams are produced. From Table 6, the use of tiles for the AI coding mode increase the bit rate from 0.6% to 6.8% when using 2 and 16 tiles, respectively. With respect to the reconstructed video quality, we can see that using tiles has no effect.

**Figure 5:**
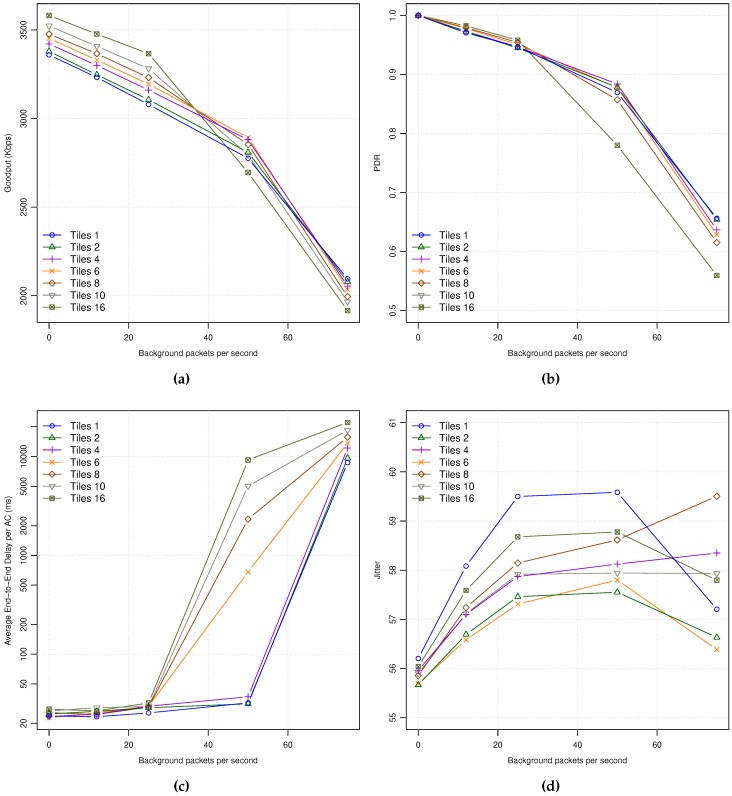
APP statistics for AI coding mode and for all the tiles per frame layouts: (**a**) GP. (**b**) PDR. (**c**) E2E-Delay. (**d**) Jitter.

In Figure 5, we show the simulation results with the same network metrics used in previous experiments. Figure 5a shows the Goodput for the different tile layouts. As expected, at low background traffic levels the curves are sorted by their corresponding bit rate. However, when background traffic increases with values greater or equal than 50 pps, and we use a high number of tiles per frame, the trend is inverted. Again, this is because the network begins to be saturated, and this situation affects more to the video sequences that require more bandwidth. The same behavior can be observed in the PDR metric shown in Figure 5b.

As shown in Figure 5c, the average E2E delay keeps similar values for all the tile number configurations at low to moderate background traffic levels, showing always slightly higher delay values when (a) increasing the number of tiles, and (b) the background traffic level increases. Notice that at 50 pps background traffic loads, only the configurations of up to 4 tiles maintain the same behavior than the one explained before at lower background traffic levels, being a symptom of entering network saturation for the AI encoding mode.

Figure 5d shows the average jitter varying the number of tiles per frame. As it can be seen, the experienced jitter is always lower on average than the one obtained with just one tile, observing better response when using a low number of tiles per frame (from 2 to 6).

**Figure 6:**
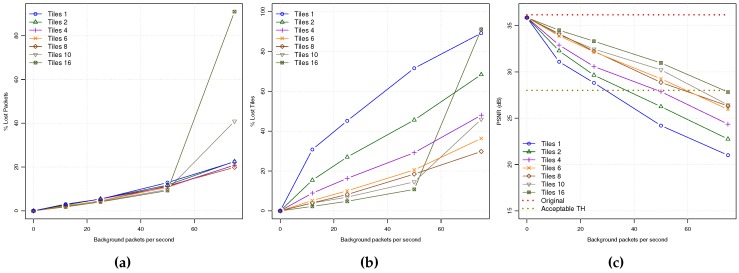
QoE statistics for AI for all the tiles per frame layouts: (**a**) PLR. (**b**) TLR. (**c**) PSNR.

From Figure 6a, we may observe a lineal increase of PLR up to the network saturation point, where a higher number of tiles always produce a lower PLR. However, in Figure 6b the TLR metric shows an interesting behavior, achieving lower TLR values when the number of tiles per frame increases. As a consequence, the video quality of the received video will be better as shown in Figure 6c. So, we can conclude that when the number of tiles per frame increases, the error resilience of the video stream also increases, obtaining acceptable video quality levels from low to moderate background traffic levels. Taking into account that the more tiles per frame the more overhead in the bit stream, several tiles of 4 or 6 would be a good trade-off.

### 5.3. Global Evaluation

**Figure 7:**
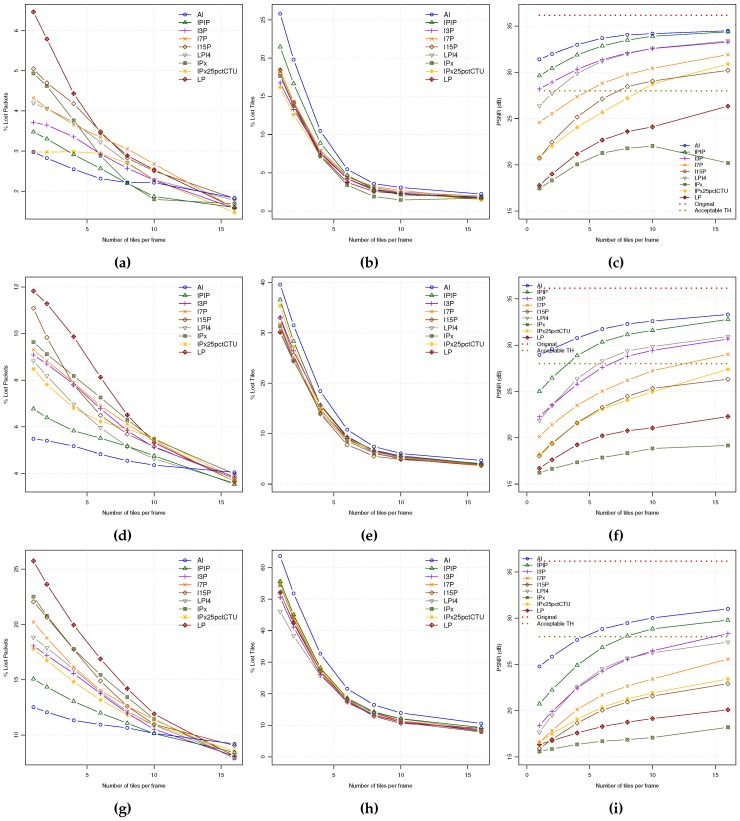
QoE statistics for all the encoding modes for all the tiles per frame layouts at different background traffic levels: (**a,b,c**) PLR, TLR, PSNR (12 pps). (**d,e,f**) PLR, TLR, PSNR (25 pps). (**g,h,i**) PLR, TLR, PSNR (50 pps).

Now, we are going to analyze the combined effect of both INTRA refresh coding modes and tiles. So, we will show the PLR, TLR and PSNR metrics for all the encoding modes as a function of the number of tiles for every coding mode for a particular background traffic level. From the previous experiments, only 12, 25, and 50 pps background traffic levels will be explored in Figure 7, since at higher traffic loads the video quality levels are unacceptable.

As it can be seen in Figure 7, there is a general behavior from low to moderate-high background traffic loads where, as the number of tiles increases (a) the PLR decreases, especially fast with the lowest bit rate coding modes (LP coding mode reduces PLR to the half by using 6 tiles); (b) the TLR also decreases at the same pace for all the coding modes, significantly reducing TLR six times for 12 pps background traffic load; and (c) the resulting PSNR also shows improvements as the number of tiles increases for all coding modes. At the other hand, when the background traffic load increases, both PLR and TLR also increases, following the same pattern as depicted before. As a consequence, the PSNR video quality decreases in general (e.g., IPIP coding mode using 6 tiles reduces PSNR in 2.5 dBs when going from 12 pps to 25 pps network load). If we define a threshold for minimum accepted video quality in 28 dBs, AI, IPIP, I3P and LPI4 will be the only coding modes that keep over that quality threshold when working with a frame partitioning of 4 tiles and low network loads (12 pps). The I7P coding mode could belong to the selected group when using 6 tiles per frame. The rest of coding modes will not satisfy the minimum quality requirement. At moderate network loads (25 pps), only AI and IPIP will be above the defined video quality threshold for 4 tiles per frame, discarding all the rest of modes. In addition, finally, at high network loads (close to saturation) only the AI coding mode will be the one that remains over the quality threshold with 6 tiles. If the number of tiles were not an issue, the IPIP coding mode could be also selected using at least 8 tiles per frame.

From this study, we may conclude that the coding modes with higher "intra refresh" degree are the ones that better stand up in front of packet losses, but they are the ones that higher network bandwidth will require. On the other hand, when using tiles, the behavior is very similar for all the coding modes, resulting in PSNR improvements. However, the consequence of using a higher number of tiles, as for high intra refreshing coding modes, is also a bit stream size increase. So, a trade-off should be defined between error resilience and bandwidth (network resources), what should be finally determined by the application requirements. We may propose a selection criteria of using a frame partitioning in no more than 6 tiles (no significant improvements are found beyond), and the coding mode that, remaining over the application quality threshold, requires the lowest bit rate.

## 6. Conclusions

In this work, we have introduced VDSF-VN, a simulation framework based on the OMNeT++ network simulator, the Veins framework and the SUMO mobility traffic simulator, which was designed to study and evaluate the robustness of a video delivery stream in vehicular ad-hoc network scenarios. Using the proposed simulation framework we analyzed different coding options, available at HEVC video encoder, to improve the robustness of the resulting video stream when it is faced with error-prone network scenarios like urban vehicular networks. We have tested different INTRA refresh coding modes and frame partitioning schemes by means of tiling, so we can determine the configurations that better performance and robustness provide at different network loads.

The experimental results show that error resilience improves as the INTRA refresh level increases. So, the best coding mode would be AI followed by IPIP, I3P, and LPI4. Notice that these encoding modes require high bit rates with respect the others, so a trade-off between error resilience and final bit rate should be defined, which strongly depends on the application requirements and the available network resources. With respect to frame partitioning, we have observed that the use of tiles has a positive impact when combined with INTRA refresh coding modes. So, acceptable video quality levels are achieved from low to moderate background traffic loads, reinforcing the properties of the INTRA refresh coding modes. Again, a trade-off should be applied since the use of tiles increases the bit stream, so the recommendation is not to use more than 6 tiles per frame since when using more tiles the improvements are, in general, negligible.

The main conclusion is that by using different INTRA refresh options combined with appropriate tile coding we will improve the protection of video streaming in VANET urban scenarios, allowing to keep acceptable video quality at high packet loss rates. We can use these error resilience techniques at encoding/decoding sides to protect the video streams. However, we need to explore additional techniques, like channel coding (FEC), the use of QoS at MAC level, and efficient error concealment, which, combined with the ones proposed here, minimize the introduced bit stream overhead and maximize the final error resilience. The final goal is focused to provide robust and efficient video streams which are able to fight against moderate to high packet network error conditions, keeping the video quality over the threshold demanded by the application. As future work, we are planning to use the available MAC QoS features, and later we will extend our error resilience architecture with packet-based FEC proposals, and advanced error concealment strategies.

## Figures and Tables

**Table 1:** Simulation parameters.

Parameter	Value
Simulation area	2000×2000 m^2^
Simulation time	340 s
Number of RSUs	3
Number of client vehicles	1
Number of background vehicles	10
Background traffic load	{0,12,25,50,75} pps
Max. speed of vehicles	14 m/s (50 km/h)

**Table 2:** PHY/MAC parameters.

Parameter	Value
Carrier frequency	5.890 GHz
Propagation model	SimpleObstacleShadowing
Bit rate	18 Mbps
Transmit power	20 mW
RX Sensitivity	−89 dBm
Communication range	510.87 m
MAC queues size	0 (infinite)

**Table 3:** Video sequence.

Parameter	Value
Name	BasketballDrill
Resolution	832×480 pixels
Duration	10 s
Length	250 frames
Rate	25 frames per second

**Table 4:** Encoding modes.

Mode	Frame layout	Description
AI	IIIIIIIII...	Every frame is an I frame (All intra)
IPIP	IP IP IP IP I...	Alternating I and P frames
I3P	IPPP IPPP I...	An I frame followed by three P frames
I7P	IPPPPPPP IPPPPPPP I...	An I frame followed by seven P frames
I15P	IPPPPPPPPPPPPPPP I...	An I frame followed by fifteen P frames
IPx	IPPPPPPPPPPPPPPP...	Similar to LP but each P frame reference the previous frame only
IPx25pctCTU	IPPPPPPPPPPPPPPP...	Similar to IPx but 25% of CTUs are forced to be Intra refreshed
LPI4	IPPP IPPP IPPP...	Similar to LP but with an I frame every four frames
LP	IPPPPPPPPPPPPPPP...	An I frame followed by only P frames (Low-delay P)

**Table 5:** QP values for achieving PSNR ≈ 36 dB (1 tiles per frame).

Mode	QP	Tiles	Bit rate (Mbps)	PSNR (dB)
AI	31	1	3.417	35.863
IPIP	30	1	2.378	36.042
I3P	30	1	1.647	35.761
I7P	29	1	1.457	36.071
I15P	29	1	1.257	35.830
IPx	28	1	1.239	35.782
IPx25pctCTU	28	1	1.797	35.812
LPI4	29	1	1.620	36.045
LP	28	1	0.959	36.160

**Table 6:** Bit rate and PSNR for AI for different tiles per frame layouts.

Mode	QP	Tiles	Bit rate (Mbps)	PSNR (dB)
AI	31	1	3.417	35.863
AI	31	2	3.438	35.866
AI	31	4	3.483	35.863
AI	31	6	3.516	35.868
AI	31	8	3.543	35.864
AI	31	10	3.578	35.862
AI	31	16	3.648	35.862

## References

[1] Garrido Abenza P.P., Piñol Peral P., P. Malumbres M., López-Granado O. Simulation Framework for Evaluating Video Delivery Services over Vehicular Networks. Proceedings of the IEEE 88th Vehicular Technology Conference (VTC-Fall).

[2] Varga A., Hornig R. An overview of the OMNeT++ Simulation Environment. Proceedings of the 1st International Conference on Simulation Tools and Techniques for Communications, Networks and Systems & Workshops.

[3] Sommer C., German R., Dressler F. (2011). Bidirectionally Coupled Network and Road Traffic Simulation for Improved IVC Analysis. IEEE Trans. Mob. Comput..

[4] Krajzewicz D., Erdmann J., Behrisch M., Bieker L. (2012). Recent Development and Applications of SUMO - Simulation of Urban MObility. Int. J. Adv. Syst. Meas..

[5] High Efficiency Video Coding (HEVC). ITU-T Recommendation H.265, 2013. http://www.itu.int/rec/T-REC-H.265.

[6] Joint Collaborative Team on Video Coding (JCT-VC) HEVC reference software HM (HEVC Test Model) and Common Test Conditions. https://hevc.hhi.fraunhofer.de.

[7] Vineeth N., Guruprasad H.S. (2013). A Survey on the Techniques Enhancing Video Streaming in VANETs. Int. J. Comput. Networking Wireless Mobile Commun..

[8] Wang Y., Wenger S., Wen J., Katsaggelos A.K. (2000). Error resilient video coding techniques. IEEE Signal Process Mag..

[9] DeBrunner V., DeBrunner L., Wang L., Radhakrishnan S. (2000). Error control and concealment for image transmission. IEEE Commun. Surv. Tutorials.

[10] Cui Z., Gan Z., Zhan X., Zhu X. (2012). Error concealment techniques for video transmission over error-prone channels: a survey. J. Comput Inf. Syst..

[11] Kung W.Y., Kim C.S., Kuo C.C.J. (2006). Spatial and Temporal Error Concealment Techniques for Video Transmission Over Noisy Channels. IEEE Trans. Circuits Syst. Video Technol..

[12] Stockhammer T., Hannuksela M.M., Wiegand T. (2003). H.264/AVC in wireless environments. IEEE Trans. Circuits Syst. Video Technol..

[13] Bandyopadhyay S.K., Wu Z., Pandit P., Boyce J.M. An Error Concealment Scheme for Entire Frame Losses for H.264/AVC. Proceedings of the 2006 IEEE Sarnoff Symposium.

[14] Girod B., Farber N. (1999). Feedback-based error control for mobile video transmission. Proc. IEEE.

[15] Kim C.S., Lee S.U. (2001). Multiple description coding of motion fields for robust video transmission. IEEE Trans. Circuits Syst. Video Technol..

[16] Hagenauer J., Stockhammer T. (1999). Channel coding and transmission aspects for wireless multimedia. Proc. IEEE.

[17] Wu J., Tan R., Wang M. (2018). Streaming High-Definition Real-Time Video to Mobile Devices With Partially Reliable Transfer. IEEE Trans. Mob. Comput..

[18] Minder L., Shokrollahi A., Watson M., Luby M., Stockhammer T. RaptorQ Forward Error Correction Scheme for Object Delivery. https://www.rfc-editor.org/rfc/pdfrfc/rfc6330.txt.pdf.

[19] Pasin M., Petracca M., Bucciol P., Servetti A., Martin J.C.D. A survey of error-concealment schemes for real-time audio and video transmissions over the Internet. Proceedings of the 4th Biennial Workshop on DSP for In-Vehicle Systems and Safety.

[20] Zhang R., Regunathan S.L., Rose K. (2000). Video coding with optimal inter/intra-mode switching for packet loss resilience. IEEE J. Sel. Areas Commun..

[21] Calafate C.T., Malumbres M.P., Manzoni P. Performance of H.264 compressed video streams over 802.11b based MANETs. Proceedings of the 24th International Conference on Distributed Computing Systems Workshops.

[22] Chen H., Zhao C., Sun M.T., Drake A. (2015). Adaptive Intra-refresh for Low-delay Error-resilient Video Coding. J. Visual Commun. Image Represent..

[23] Klaue J., Rathke B., Wolisz A. (2003). EvalVid – A Framework for Video Transmission and Quality Evaluation.

[24] Ke C., Shieh C., Hwang W., Ziviani A. (2008). An Evaluation Framework for More Realistic Simulations of MPEG Video Transmission. J. Inf. Sci. Eng..

[25] Saladino D., Paganelli A., Casoni M. (2013). A tool for multimedia quality assessment in NS3: QoE Monitor. Simul. Modell. Pract. Theory.

[26] Rosário D., Zhao Z., Silva C., Cerqueira E., Braun T. An OMNeT++ Framework to Evaluate Video Transmission in Mobile Wireless Multimedia Sensor Networks. Proceedings of the 6th International ICST Conference on Simulation Tools and Techniques.

[27] Pediaditakis D., Tselishchev Y., Boulis A. Performance and scalability evaluation of the Castalia wireless sensor network simulator. Proceedings of the 3rd International ICST Conference on Simulation Tools and Techniques.

[28] Nightingale J., Wang Q., Grecos C. (2012). HEVStream: a framework for streaming and evaluation of high efficiency video coding (HEVC) content in loss-prone networks. IEEE Trans. Consum. Electron..

[29] Garrido Abenza P.P., P. Malumbres M., Piñol Peral P. GatcomSUMO: A Graphical Tool for VANET Simulations Using SUMO and OMNeT++. Proceedings of the 2017 SUMO User Conference.

[30] Wang Z., Simoncelli E.P., Bovik A.C. Multiscale structural similarity for image quality assessment. Proceedings of the Thirty-Seventh Asilomar Conference on Signals, Systems Computers.

[31] Haklay M.M., Weber P. (2008). OpenStreetMap: User-Generated Street Maps. IEEE Pervasive Comput..

[32] Krauss S. (1998). Microscopic Modeling of Traffic Flow: Investigation of Collision Free Vehicle Dynamics. Ph.D. Thesis.

[33] Misra K., Segall A., Horowitz M., Xu S., Fuldseth A., Zhou M. (2013). An Overview of Tiles in HEVC. IEEE J. Sel. Top. Sign. Proces..

[34] Seeling P., Reisslein M. (2012). Video Transport Evaluation With H.264 Video Traces. IEEE Commun. Surv. Tutorials.

[35] Wang Y., Sanchez Y., Schierl T., Wenger S., Hannuksela M. RTP Payload Format for High Efficiency Video Coding. https://www.rfc-editor.org/rfc/pdfrfc/rfc7798.txt.pdf.

